# Interplay between genetic risk and built neighborhood conditions as predictor of BMI across the transition into adulthood

**DOI:** 10.1002/oby.24213

**Published:** 2025-01-19

**Authors:** Marthe de Roo, Catharina A. Hartman, Alfred Wagtendonk, Hans W. Hoek, Jeroen Lakerveld, Tina Kretschmer

**Affiliations:** ^1^ Faculty of Behavioral and Social Sciences, Department of Pedagogy and Educational Sciences University of Groningen Groningen the Netherlands; ^2^ Interdisciplinary Center Psychopathology and Emotion Regulation (ICPE), University of Groningen University Medical Center Groningen Groningen the Netherlands; ^3^ Department of Epidemiology and Data Science, Amsterdam Public Health Research Institute, Amsterdam University Medical Center Vrije Universiteit Amsterdam Amsterdam the Netherlands; ^4^ Upstream Team, Amsterdam University Medical Center Vrije Universiteit Amsterdam Amsterdam the Netherlands; ^5^ Parnassia Psychiatric Institute The Hague the Netherlands; ^6^ Department of Psychiatry University of Groningen, University Medical Center Groningen Groningen the Netherlands; ^7^ Department of Epidemiology Columbia University New York New York USA

## Abstract

**Objective:**

We examined BMI development across changes in the built environment during the transition from adolescence to young adulthood and explored the moderating role of genetic risk.

**Methods:**

We used longitudinal data from individuals aged 16 to 25 years in the TRacking Adolescents' Individual Lives Survey (TRAILS) that we linked to built environment data for 2006, 2010, and 2016 from the Geoscience and Health Cohort Consortium (GECCO). We fitted a latent growth model of BMI and examined associations of changes in fast‐food restaurant density and walkability with changes in BMI (*n* = 2735), as well as interactions of changes in fast‐food restaurant density and walkability with genetic risk (*n* = 1676).

**Results:**

Changes in fast‐food restaurant density (e.g., Δ2010–2006: *β* = −0.04, 95% CI: −0.11 to 0.03) and walkability (e.g., Δ2010–2006: *β* = −0.05, 95% CI: −0.14 to 0.05) were not associated with BMI changes. Additionally, genetic risk did not moderate these associations.

**Conclusions:**

We found limited evidence that moving to neighborhoods with higher fast‐food restaurant density or less walkability was associated with BMI changes or that genetic risk moderated these associations. Our findings suggest that associations between the built environment and BMI changes during the transition into young adulthood are likely small.

**Study Importance**

**What is already known?**

- Genetic influence on BMI increases from childhood through adolescence.
- Previous studies have suggested that “obesogenic” environments facilitate a sedentary lifestyle and access to unhealthy foods; however, robust evidence linking the built environment to weight‐related outcomes is lacking.

**What does this study add?**

- Moving to areas with higher fast‐food restaurant density or less walkability during the transition to young adulthood was not associated with changes in BMI in our sample.
- We found limited evidence that genetic risk moderated the associations between changes in fast‐food restaurant density and walkability and BMI development.

**How might these results change the direction of research?**

- As the link between obesogenic neighborhood environments and weight gain during the transition from adolescence into young adulthood is likely small, future research should focus on the choices that individuals make within those neighborhoods to provide a more nuanced representation of the association between the built environment and weight.

## INTRODUCTION

The increasing prevalence of youth with overweight has made understanding its causes highly important [1]. Individual differences in body mass index (BMI) are shaped by the complex interplay between genes and environment whereby young people's choices and responses to changes in their environment are influenced by their genetic makeup. During adolescence, food decisions become less dependent on parents, and individuals start making their own dietary and lifestyle choices. Once they have moved out of the parental home, adolescents and young adults are even more independent in shaping their health‐related habits. The transition from adolescence to young adulthood is therefore a critical phase during which gene–environment interactions (G × E) might substantially influence variation in BMI outcomes. For instance, those at higher genetic risk for developing overweight seem more prone to cravings for foods that are high in fat and sugar that influence their environmental choices [2].

Thus far, our understanding of genetic and environmental contributions to weight has largely relied on twin studies [3]. These studies have demonstrated that genetic influence on BMI varies across development, with heritability estimates increasing during childhood and adolescence and peaking at the onset of adulthood [4]. More recently, findings from twin samples have been confirmed by molecular genetic studies that have focused on individual differences in DNA structure and variation in traits, including overweight [5, 6].

Next to genetic factors, “obesogenic” environments that facilitate a sedentary lifestyle and consumption of unhealthy foods might drive the rising prevalence of overweight [7]. Studies into socioecological determinants of overweight have therefore focused on built environments, including (un)healthy residential neighborhood characteristics [8]. That is, people likely engage in less physical activity in neighborhoods that are unsuitable for walking, which poses a risk for developing high BMI values [9]. Similarly, ease of access to stores and restaurants with high‐calorie meals can lead to increased consumption of unhealthy foods and subsequent weight gain [10]. Although there is some evidence linking the physical activity environment, particularly walkability, to weight, most studies on the built environment and weight‐related outcomes have thus far found limited evidence [11, 12, 13, 14]. For the food environment, the strongest evidence was found for fast‐food restaurant exposure, although even within this domain findings remain inconclusive [11]. Associations might differ across the degree of neighborhood deprivation, with increased exposure to fast‐food restaurants having only been linked to BMI in more deprived areas [15, 16].

Individual differences in genetic predisposition for overweight might be another influence on who remains lean and who gains weight in an obesogenic environment. However, only limited G × E research exists on the role of the wider food and physical activity environments that shape individual behaviors. Studies in adults have suggested that genetic factors influence susceptibility to obesogenic neighborhood characteristics, with stronger genetic influence in more deprived neighborhoods [17, 18], less walkable neighborhoods [19], and neighborhoods with more fast‐food restaurants [20, 21]. However, much of this research has relied on twin designs rather than genotype information to estimate genetic influence [17, 19]. In contrast to studies using twin samples, molecular genetic designs directly measure genetic risk, and analyses can be conducted in any genotyped sample. By applying a G × E framework, we can elucidate which features of the neighborhood environment interact with genetic risk to influence overweight. An understanding of which genetic and built environment risk factors are associated with higher BMI can contribute to interventions that support young people in developing healthy dietary and lifestyle habits and might also inform policy responses or neighborhood‐level interventions.

Longitudinal studies that have explored the interplay between genes and environment on BMI in adolescence and across the transition into young adulthood are currently lacking. This is a problem because genetic factors associated with BMI increasingly influence weight during adolescence and beyond [4], reflecting young people's growing autonomy to select and create environments in line with their genetic predispositions [22]. In other words, a developmental approach is necessary to study how genes and the built environment interact to shape BMI during this transition.

We used a polygenic score for BMI to examine BMI development across changes in the built environment during the transition into young adulthood and to explore genetic moderation. A polygenic score represents an individual's genetic predisposition for a phenotype, derived from relevant genome‐wide association study data [23]. We used longitudinal data spanning 9 years from a large‐scale cohort and objective environmental measurements [11, 12]. We hypothesized that individuals moving to neighborhoods with higher fast‐food restaurant density and less walkability increase more in BMI and that these associations are particularly strong for those at high genetic risk for overweight. We controlled for age, sex, family socioeconomic status, mental health, and whether participants still lived at home, as these might influence BMI changes [6]. Additionally, we controlled for neighborhood socioeconomic status and urban density to isolate the influence of fast‐food restaurant density and walkability on BMI changes independent of broader socioeconomic and urban contextual factors.

## METHODS

### Participants

We used data from six waves of the TRacking Adolescents' Individual Lives Survey (TRAILS), an ongoing longitudinal study of Dutch adolescents recruited in the north of the Netherlands with bi‐ or triennial follow‐up assessments [24]. TRAILS consists of a population and high‐risk sample: The population sample was recruited through community registers and primary schools, whereby, out of 2395 children approached for enrollment, 2229 (50.8% girls) agreed to participate. Data collection for the initial assessment wave (T1) took place in 2001 and 2002 (mean age, 11.1 years), the second wave (T2) in 2003 and 2004 (mean age, 13.6 years), the third wave (T3) in 2006 and 2007 (mean age, 16.3 years), the fourth wave (T4) in 2008 to 2010 (mean age, 19.1 years), the fifth wave (T5) in 2012 and 2013 (mean age, 22.3 years), and the sixth wave (T6) in 2016 (mean age, 25.7 years).

The high‐risk sample was set up in 2004 and included 543 children selected based on having been in contact with mental health services before age 11 years, indicating a higher risk of mental health problems (response rate, 43%). This sample included more boys (66%), in line with sex ratios for the most common childhood psychopathologies. Initial data collection occurred in 2004 to 2005 (mean age, 10.9 years), with follow‐up assessments every 2 or 3 years, lagging approximately one assessment wave behind the population sample: T2 in 2006 to 2007 (mean age, 13.0 years), T3 in 2009 to 2011 (mean age, 15.9 years), T4 in 2012 to 2014 (mean age, 19.1 years), and T5 in 2015 to 2017 (mean age, 22.0 years).

Retention rates ranged between 73% and 96% for the population sample and between 73% and 85% for the high‐risk sample. Ethics approval was obtained from the Dutch Central Committee on Research Involving Human Subjects before the start, and written consent was obtained from participants and parents. See online Supporting Information Appendix A for attrition analyses for the data used here.

### Data linkage

We linked TRAILS participants' data from ages 16, 19, and 25 years (corresponding to the years 2006, 2010, and 2016) to built environment data collected by the Geoscience and Health Cohort Consortium (GECCO) [25, 26]. We selected these time points to align BMI measurements with periods when built environment data were available, focusing on the developmental phase when participants transitioned from living with their parents to living independently. When built environment data were not available for one or more of these time points, we obtained the data for the closest available year to our intended time frame. We matched built environment variables to the TRAILS assessment wave conducted either in the same year or in the closest corresponding year. Given that the high‐risk sample lagged behind by one assessment wave compared with the population sample, the specific TRAILS assessment wave to which the built environment data was linked differed between the two samples. Built environment data were matched with participants' residential addresses (i.e., six‐digit postal code and house number), six‐digit postal codes, or four‐digit postal codes, depending on the available spatial scale of the built environment variables. Table 1 provides an overview of the included built environment data and corresponding TRAILS waves. Information on the TRAILS waves that were used to construct the variables for each time point is reported in Table 2. In line with prior research conducted in the Netherlands, exposures on address level were calculated within circular buffers of 1000 m around participants' homes [21].

**TABLE 1:** Overview of data availability for built environment variables as collected by GECCO and corresponding TRAILS waves.

			Corresponding TRAILS wave				Total no.
Built environment variables			Population cohort		High‐risk cohort		
Exposure	Spatial scale	Year	Assessment wave	Available no.	Assessment wave	Available no.	
Fast‐food restaurant density	Address	2006	T3 (2005–2007)	1703	T2 (2006–2007)	445	2148
		2010	T4 (2008–2010)	1809	T3 (2009–2011)	401	2210
		2016	T6 (2016–2017)	1458	T5 (2015–2017)	369	1827
Walkability	Address	2006	T3 (2005–2007)	1703	T2 (2006–2007)	445	2148
		2010	T4 (2008–2010)	1809	T3 (2009–2011)	401	2210
		2015	T6 (2016–2017)	1458	T5 (2015–2017)	369	1827
Neighborhood socioeconomic status	4‐digit postal code	2006	T3 (2005–2007)	1742	T2 (2006–2007)	454	2196
		2010	T4 (2008–2010)	1851	T3 (2009–2011)	406	2257
		2016	T6 (2016–2017)	1500	T5 (2015–2017)	375	1875
Urban density (addresses/km^2^)	6‐digit postal code	2006	T3 (2005–2007)	1732	T2 (2006–2007)	449	2181
		2011	T4 (2008–2010)	1844	T3 (2009–2011)	405	2249
		2016	T6 (2016–2017)	1492	T5 (2015–2017)	375	1867

Abbreviations: GECCO, Geoscience and Health Cohort Consortium; TRAILS, TRacking Adolescents' Individual Lives Survey.

**TABLE 2:** Descriptive statistics of individual‐level variables and built environment data.

	Corresponding TRAILS wave^a^		Full sample (*N* = 2735)			Participants with genetic data (*n* = 1676)		
	Population cohort	High‐risk cohort	Mean (SD) or percentage	*n* total	% missing	Mean (SD) or percentage	*n* total	% missing
BMI								
2006	T3 (2005–2007)	T2 (2006–2007)	20.80 (3.43)	1991	27.2%	20.76 (3.35)	1599	4.6%
2010	T4 (2008–2010)	T3 (2009–2011)	22.62 (3.89)	1916	29.9%	22.61 (3.80)	1440	14.1%
2016	T6 (2016–2017)	T5 (2015–2017)	24.16 (4.44)	1565	42.8%	24.16 (4.41)	1184	29.4%
Sex	T1 (2001–2002)	T1 (2004–2005)	47.4% female	2735	0.0%	48.2% female	1676	0.0%
Age								
2006	T3 (2005–2007)	T2 (2006–2007)	15.59 (1.53)	2249	17.8%	15.55 (1.51)	1661	0.9%
2010	T4 (2008–2010)	T3 (2009–2011)	18.51 (1.36)	2266	17.1%	18.40 (1.37)	1585	5.4%
2016	T6 (2016–2017)	T5 (2015–2017)	24.93 (1.60)	1986	27.4%	24.88 (1.60)	1451	13.4%
Family SES	T1 (2001–2002)	T1 (2004–2005)	−0.05 (0.79)	2691	1.6%	0.08 (0.76)	1664	0.7%
Mental health	T3 (2005–2007)	T2 (2006–2007)	0.23 (0.21)	1921	29.8%	0.22 (0.21)	1473	12.1%
Living situation								
2006	T3 (2005–2007)	T2 (2006–2007)	0.5% living away from home	2735	0.0%	0.6% living away from home	1676	0.0%
2010	T4 (2008–2010)	T3 (2009–2011)	23.1% living away from home	2677	2.1%	24.1% living away from home	1659	1.0%
2016	T6 (2016–2017)	T5 (2015–2017)	78.8% living away from home	2033	25.7%	79.6% living away from home	1472	12.2%
Neighborhood SES								
2006	T3 (2005–2007)	T2 (2006–2007)	−0.19 (1.07)	2196	19.8%	−0.15 (1.05)	1634	2.5%
2010	T4 (2008–2010)	T3 (2009–2011)	−0.85 (1.42)	2257	17.5%	−0.81 (1.38)	1579	5.8%
2016	T6 (2016–2017)	T5 (2015–2017)	−0.86 (1.44)	1875	31.4%	−0.83 (1.41)	1389	17.1%

*Note*: The polygenic score for BMI (*n* = 1676), change in fast‐food restaurant density (*n* ranges from 1827 to 2210), change in walkability (*n* ranges from 1827 to 2210), and urban density (*n* ranges from 1867 to 2181) were standardized and are therefore not included in this table.

Abbreviations: SES, socioeconomic status; TRAILS, TRacking Adolescents' Individual Lives Survey.

^a^ The TRAILS assessment waves listed correspond to the data collection periods used to construct the individual‐level variables for 2006, 2010, and 2016.

### Measures

#### BMI

Weight and height were measured by trained research assistants at T2 to T5 using a calibrated scale (Seca 770, Seca GmbH) and a stadiometer (Seca 214, Seca GmbH). At T6, weight and height were self‐reported. BMI was calculated by dividing weight by height squared (kilograms divided by meters squared). In order to address the time lag of one assessment wave in the high‐risk sample compared with the population sample, we created BMI variables based on assessment year (2006, 2010, and 2016) rather than assessment wave, corresponding to BMI at ages 16, 19, and 25 years, respectively.

#### Change in fast‐food restaurant density

Data for fast‐food restaurant density came from Locatus, a Dutch company that collects information on the locations and types of retailers in Europe. We used Locatus data on the kernel density of fast‐food restaurants within a 1000‐m radius of each residential address for the years 2006, 2010, and 2016. Kernel densities are used to create a weighted availability or intensity measure in which the distances to all food retailers within the search radius are reflected for every (virtual) grid cell location. The application of kernel density estimation implies the attribution of location‐specific density values to the address locations of individuals with, e.g., higher density values close to food retailers clustered together [27, 28]. In order to assess changes in fast‐food restaurant density between two time points, we computed difference scores by subtracting each time point's score from the previous one, resulting in the following two difference scores: one reflecting fast‐food restaurant density differences between 2006 (age 16 years) and 2010 (age 19 years) and the other between 2010 (age 19 years) and 2016 (age 25 years). Positive scores indicate an increase in fast‐food restaurant density between time points.

#### Change in walkability

The walkability index was based on seven different spatial components, including population density, density of retail and service destinations, land‐use mix, street connectivity, green space, sidewalk surface area, and public transport density [29]. All components were summed and normalized to a continuous walkability index ranging from 0 to 100, with higher scores indicating greater walkability. The index was not available for 2016; therefore, we included the indices for the years 2006, 2010, and 2015. We measured changes in walkability over time by computing the following two difference score variables: one capturing walkability differences between 2006 (age 16 years) and 2010 (age 19 years) and the other between 2010 (age 19 years) and 2015 (age 25 years). Positive scores reflect an increase in walkability between two time points.

#### Polygenic score for BMI

Genetic data were available for *n* = 1676 participants. Details on DNA extraction and participant exclusion are provided in online Supporting Information Appendix B. The polygenic score for BMI was computed using summary statistics of adult BMI from the Genetic Investigation of Anthropometric Traits Consortium (*N* = ~700,000) [30]. Adult‐derived polygenic scores for BMI can predict BMI during adolescence with similar accuracy to BMI in adults [5]. Additionally, genome‐wide association studies based on adult BMI values involve much larger sample sizes compared with those focusing on child BMI, thereby providing more robust estimates of genetic risk. TRAILS data were excluded from the summary statistics using R package MetaSubtract (version 1.60) [31]. We used LDpred2‐auto to calculate the polygenic score, which automatically estimates the single‐nucleotide polymorphism‐heritability (*h* ^2^) and the proportion of causal variants (*p*) without the need for a validation dataset [32]. Only HapMap3+ variants were included (*n* = 920,337), which passed rigorous quality control and provide a good coverage of the whole genome [32]. We used the linkage disequilibrium reference panel based on European individuals of the UK Biobank provided by the developers of LDpred2.

#### Covariates

We controlled for age at baseline, sex, cohort, family socioeconomic status, living situation, baseline mental health, neighborhood socioeconomic status, and urban density. Family socioeconomic status was measured at T1 using both parents' educational and occupational levels and family income. Educational level was assessed in five categories, ranging from elementary to university education. Occupational level was based on the International Standard Classification of Occupations [33]. Family net income was measured on a scale ranging from 1 (<€680 per month) to 9 (>€3857 per month). Socioeconomic status was calculated as the mean of these standardized five items (Cronbach *α* = 0.84). We created variables representing living situation (1 = living away from home and 2 = living in family home) for 2006 (age 16 years), 2010 (age 19 years), and 2016 (age 25 years) based on whether participants' addresses matched those of their parents. Mental health at baseline was assessed by parents using the Internalizing Problems subscale of the Child Behavior Checklist (Cronbach *α* = 0.87). Neighborhood socioeconomic status was calculated for 2006, 2010, and 2016 by the Netherlands Institute of Social Research and indicates the relative social status of a four‐digit postal code district in the Netherlands compared with others [34]. Scores were based on population averages of education, income, and position in the labor market. Scores were standardized to have an average of zero, with higher scores indicating higher socioeconomic status. Urban density was determined for 2006, 2011, and 2016 using residential address density data provided by Statistics Netherlands and defined as the average number of residential addresses within a 1000‐m radius.

### Analytic strategy

We specified a latent growth model of BMI without covariates using Mplus version 8.6 (Muthén & Muthén). We estimated an intercept‐only model and a model with both intercept and linear slope with average time in years since the first time point as metric of time, resulting in the following time coding: zero for age 16 years (2006, T1), four for age 19 years (2010, T2), and ten for age 25 years (2016, T3). We evaluated model fit using the Akaike information criterion (AIC; smaller values indicate better fit), χ^2^ test (nonsignificant values indicate that the model fits the data), comparative fit index (CFI; values closer to 1 indicate better fit), Tucker‐Lewis index (TLI; values closer to 1 indicate better fit), root‐mean‐square error of approximation (RMSEA; values closer to 0 indicate better fit), and standardized root‐mean‐square residual (SRMR; values closer to 0 indicate better fit).

Next, we first tested for main environmental effects by entering the fast‐food restaurant density difference scores (Model 1A) and the walkability difference scores (Model 2A) as time‐varying covariates to predict BMI changes. Second, the polygenic score for BMI, along with interaction terms between the polygenic score for BMI and fast‐food restaurant density difference scores (Model 1B) and walkability difference scores (Model 2B), was added as predictors of slope to test for G × E. All models were adjusted for sex, age at baseline, cohort, family socioeconomic status, and baseline mental health as time‐invariant covariates and living situation, neighborhood socioeconomic status, and urban density as time‐varying covariates. Only time‐invariant covariates and time‐varying covariates measured at baseline were added as predictors of intercept. In models that included the polygenic score, we added 20 principal components of the genetic data to control for population stratification. All predictors were standardized to improve interpretability. Missing values were handled using multiple imputation, resulting in a final analytic sample of *N* = 2735 for analyses excluding the polygenic score for BMI and *n* = 1676 for analyses including the polygenic score. Using SPSS version 28.0.0.0 (IBM Corp.), we generated 20 datasets with 50 iterations using the fully conditional specification method (predictive mean matching). Analyses were conducted in the imputed datasets in Mplus, and pooled estimates are reported. As a sensitivity check, we repeated the analyses using 500‐ and 1500‐m buffers for fast‐food restaurant density and 500‐ and 1650‐m buffers for walkability [35]. Variations in buffer sizes were due to differences in availability.

## RESULTS

Table 2 contains descriptive statistics for the full sample (*N* = 2735) and for participants with genetic data (*n* = 1676). The polygenic score for BMI explained up to 13.0% of BMI variance in TRAILS participants. Compared with those without genetic data, participants with genetic data available were older at baseline (*t*_2247_ = −2.04; *p* = 0.04; Cohen *d* = −0.10), had higher family socioeconomic status (*t*_2689_ = 11.16; *p* < 0.001; Cohen *d* = 0.44), resided in neighborhoods with higher socioeconomic status at ages 16 years (*t*_2194_ = 3.19; *p* = 0.001; Cohen *d* = 0.15) and 19 years (*t*_2255_ = 2.04; *p* = 0.04; Cohen *d* = 0.09), lived in more rural areas at age 16 years (*t*_2179_ = −2.06; *p* = 0.04; Cohen *d* = −0.10), and lived in more urban areas at age 25 years (*t*_1865_ = 2.46; *p* = 0.01; Cohen *d* = 0.13). Correlations are presented in Figure S1.

### Latent growth analyses of BMI

Model fit indices for the latent growth analyses of BMI can be found in Table S1. We first fitted an intercept‐only model to establish baseline fit. Subsequently, we estimated a model with linear slope. The model comparison showed that adding a linear slope improved the fit over the intercept‐only model (Δ*χ* ^2^_3_ = 1846.53; *p* < 0.001), and the linear model generally demonstrated reasonable fit to the data (AIC = 40,543.06, CFI = 0.96, TLI = 0.87, RMSEA = 0.17, and SRMR = 0.05) [36]. Table 3 shows parameter estimates of the linear model, and a plot depicting the predicted trajectory of BMI is included in Figure S2.

**TABLE 3:** Parameter estimates from the latent growth model of linear change in BMI.

	Estimate	SE	*p* value
Mean intercept	20.92	0.08	<0.001
Mean slope	0.35	0.01	<0.001
Intercept variance	13.42	0.64	<0.001
Slope variance	0.11	0.01	<0.001
Covariance (intercept, slope)	−0.26	0.06	<0.001

*Note*: *n* = 2735. All path estimates shown are unstandardized except for the correlation between intercept and slope.

### Change in fast‐food restaurant density and change in walkability as predictors of BMI change

Moving to a neighborhood with higher fast‐food restaurant density was not associated with a steeper BMI increase (Table 4, Model 1A). Similarly, moving to a less walkable neighborhood was not associated with BMI change (Table 5, Model 2A).

**TABLE 4:** Standardized coefficients from models predicting BMI increase from the fast‐food restaurant density difference scores, PGS_BMI_, and the fast‐food restaurant density difference scores × PGS_BMI_.

		Intercept		Slope	
Model	Predictor	*β* (SE)	95% CI	*β* (SE)	95% CI
1A. Main effect fast‐food restaurant density difference score (*n* = 2735)	Fast‐food restaurant density (Δ2010–2006)			−0.04 (0.04)	(−0.11 to 0.03)
	Fast‐food restaurant density (Δ2016–2010)			−0.01 (0.04)	(−0.08 to 0.07)
1B. Fast‐food restaurant density difference score × PGS_BMI_ (*n* = 1676)	Fast‐food restaurant density (Δ2010–2006)			−0.03 (0.04)	(−0.11 to 0.04)
	Fast‐food restaurant density (Δ2016–2010)			0.01 (0.04)	(−0.06 to 0.09)
	PGS_BMI_	0.31 (0.02)*	(0.27 to 0.35)	0.12 (0.03)*	(0.07 to 0.18)
	Fast‐food restaurant density (Δ2010–2006) × PGS_BMI_			−0.02 (0.03)	(−0.08 to 0.03)
	Fast‐food restaurant density (Δ2016–2010) × PGS_BMI_			−0.01 (0.03)	(−0.06 to 0.04)

*Note*: Associations were adjusted for sex, age at baseline, cohort, family socioeconomic status, baseline mental health, and 20 principal components (Model B) as time‐invariant covariates and living situation, neighborhood socioeconomic status, and urban density as time‐varying covariates.

Abbreviation: PGS_BMI_, polygenic score for BMI.

* *p* < 0.05.

**TABLE 5:** Standardized coefficients from models predicting BMI increase from the walkability difference scores, PGS _BMI_, and the walkability difference scores × PGS_BMI_.

		Intercept		Slope	
Model	Predictor	*β* (SE)	95% CI	*β* (SE)	95% CI
2A. Main effect walkability difference score (*n* = 2735)	Walkability (Δ2010–2006)			−0.05 (0.05)	(−0.14 to 0.05)
	Walkability (Δ2015–2010)			0.00 (0.04)	(−0.08 to 0.08)
2B. Walkability difference score × PGS_BMI_ (*n* = 1676)	Walkability (Δ2010–2006)			−0.05 (0.05)	(−0.15 to 0.05)
	Walkability (Δ2015–2010)			0.01 (0.05)	(−0.08 to 0.10)
	Polygenic score for BMI	0.32 (0.02)*	(0.28 to 0.36)	0.11 (0.03)*	(0.06 to 0.17)
	Walkability (Δ2010–2006) × PGS_BMI_			−0.02 (0.03)	(−0.08 to 0.04)
	Walkability (Δ2015–2010) × PGS_BMI_			0.01 (0.03)	(−0.05 to 0.07)

*Note*: Associations were adjusted for sex, age at baseline, cohort, family socioeconomic status, baseline mental health, and 20 principal components (Model B) as time‐invariant covariates and living situation, neighborhood socioeconomic status, and urban density as time‐varying covariates.

Abbreviation: PGS_BMI_, polygenic score for BMI.

* *p* < 0.05.

### Genetic risk and G × E interactions as predictors of BMI change

Genetic risk for higher BMI was a moderate predictor of BMI at age 16 years (e.g., *β* = 0.31, 95% confidence interval [CI]: 0.27–0.35; Table 4, Model 1B). When converted back to the original BMI scale, this indicated that a one standard deviation (SD) increase in the polygenic score for BMI was associated with a 1.10‐kg/m^2^ higher BMI at age 16 years. This association became stronger over time (*β* = 0.12, 95% CI: 0.07–0.18; Table 4, Model 1B, and *β* = 0.11, 95% CI: 0.06–0.17; Table 5, Model 2B), indicating that, for each SD increase in genetic risk, the annual rate of change in BMI was, on average, 0.04 kg/m^2^ higher. Genetic risk did not moderate the influence of moving to neighborhoods with higher fast‐food restaurant density or more walkable neighborhoods on BMI change (Models 1B and 2B). Sensitivity analyses produced results consistent with the main analyses (Tables S2–S5), and analyses based on complete cases showed results that were highly similar to those obtained from the imputed data (Tables S6 and S7).

## DISCUSSION

The transition from adolescence to young adulthood is an important period to study factors contributing to weight gain, as young people become more independent in making dietary and lifestyle choices. We leveraged information on adolescents' relocations during this transition to examine whether BMI development was associated with changes in the environment, specifically changes in fast‐food restaurant density and walkability. Furthermore, we examined the interplay of genes and the built environment on BMI change.

There was little evidence to suggest that participants who moved to neighborhoods with higher fast‐food restaurant density experienced greater BMI increases than those who moved to neighborhoods with fewer fast‐food restaurants. Neighborhoods with high fast‐food restaurant density are likely situated in urbanized settings that provide a large variety of options for both food and physical activity, including healthier choices. In the major cities of the Netherlands, neighborhoods located in city centers not only offer access to unhealthy food but also provide extensive opportunities for various forms of exercise [11]. Exclusively focusing on fast‐food restaurant density might thereby oversimplify the environment, which contains features that promote and discourage healthy behaviors [37].

Consistent with findings from twin studies [3, 4], we found moderate genetic influence on BMI change that increased with age. Parents are an important environmental source of variation in BMI in childhood; however, their influence decreases during the transition into young adulthood when individuals increasingly select and create their own environments. Such choices may, in part, be driven by genes change people's ability to actively select specific neighborhoods to live in, genetic variants that affect BMI might also shape broader life decisions such as attending college or choosing to remain in the parental home.

Overall, genetic risk did not appear to alter the association between changes in fast‐food restaurant density and BMI change, although G × E has been reported previously [20, 21]. Interestingly, a study that investigated both cross‐sectional and longitudinal associations found a stronger association between fast‐food restaurant exposure and BMI, but not BMI change, in young adults with higher genetic risk [21]. This suggests that interactions between genetic factors and the built environment might not be stable over time.

Similar to the results for fast‐food restaurant density, we found little evidence that moving to less walkable neighborhoods was associated with greater BMI increases compared with moving to more walkable areas. This contradicts previous research on longitudinal associations between neighborhood walkability and weight‐related outcomes that suggested that living in walkable neighborhoods might protect against weight gain by encouraging physical activity [39].

We also found limited support for the hypothesis that the association between moving to less walkable areas and BMI increases was stronger among individuals with higher genetic risk. Although some previous research has suggested that individuals with higher genetic risk might be more sensitive to exposure to obesogenic environments [17, 18, 19, 20, 21], these studies have used cross‐sectional designs and may only provide snapshots of the associations. Moreover, most of these studies have relied on twins to examine G × E [17, 18, 19, 20]. The discrepancy between findings from twin and polygenic score studies emphasizes the need for diverse approaches to examine G × E interplay associated with BMI development.

Taken together, our findings indicate that associations between the built environment and the development of overweight are likely small. However, even small effects can be relevant for population health when many people are exposed [40]. The importance of the neighborhood environment could also vary across life stages, with individual‐level factors such as parenting practices and individual routines playing a more pronounced role during the transition into young adulthood. Future studies may benefit from assessing the choices that individuals make within their neighborhoods and the reasons behind these choices. Understanding where young people choose to eat, whether they use nearby sports facilities, and their engagement in other lifestyle behaviors can provide a more nuanced representation of the associations between the built environment and weight.

Despite strengths such as the use of longitudinal data, the use of a polygenic score for BMI, and objective measurements of the built environment, there were some limitations. First, environmental data were not available for all TRAILS assessment waves, limiting our analysis to three time points aligned with the available environmental data. Second, weight and height were self‐reported at T6. Given that individuals tend to underestimate their weight, the reliance on self‐reports may have biased our results [41]. However, at T5, both self‐reports and objective measurements were collected, and the high correlation of 0.95 between the two indicated only a minimal discrepancy. Third, genetic data were not available for the full sample, which reduced the sample size and statistical power for analyses involving the polygenic score for BMI, particularly for the G × E analyses, which typically require large samples [42]. Additionally, participants with genetic data had higher socioeconomic status than participants without genetic data, which might have led to an attenuation of reported estimates. Fourth, measures of the built environment were based on where participants lived but ignored where they spent their time. For example, patterns may be more pronounced when examining the built environment surrounding schools or workplaces [43]. Fifth, the modifiable aggregation of geographical units can introduce bias [44]. To reduce the impact of arbitrary spatial aggregation, we assessed built environment exposures within buffers around participants' homes rather than aggregating data into areas such as postal code zones. We also conducted sensitivity analyses using other buffer sizes and found no impact on the results [35]. A final limitation is the potential for bidirectional influence, in which changes in BMI could also impact built environment factors.

## CONCLUSION

We examined how changes in the built environment, specifically changes in fast‐food restaurant density and walkability, in interplay with genetic risk influenced BMI development during the transition to young adulthood. Changes in fast‐food restaurant density or walkability did not show a clear association with BMI development, nor did genetic risk moderate responses to changes in the built environment. Only genetic risk was moderately associated with increases in BMI. Overall, our findings suggest that the development of overweight during the transition into young adulthood is influenced by genetic factors, but we did not find convincing evidence for an influence of “obesogenic” neighborhoods.

## FUNDING INFORMATION

This research is part of the TRacking Adolescents' Individual Lives Survey (TRAILS). Participating centers of TRAILS include various departments of the University Medical Center and University of Groningen, the University of Utrecht, the Radboud University Medical Center, and the Parnassia Psychiatric Institute, which are all located in the Netherlands. TRAILS has been financially supported by various grants from the Netherlands Organization for Scientific Research (NWO), the Netherlands Organisation for Health Research and Development (ZonMW), GB MaGW, the Dutch Ministry of Justice, the European Science Foundation, the European Research Council, BBMRI.nl, and the participating universities. Tina Kretschmer was supported by a Starting Grant and a Consolidator Grant from the European Research Council (grant numbers 757364 and 101087395). Which was financially supported by the NWO, ZonMW, and Amsterdam University Medical Center.

## CONFLICT OF INTEREST STATEMENT

The authors declared no conflicts of interest.

## Supporting information


**Data S1.** Supporting Information.
