# Supplementary material for: Interplay between genetic risk and built neighborhood conditions as predictor of BMI across the transition into adulthood
Source: Obesity (Silver Spring). 2025 Jan 19;33(2):385–94. doi: 10.1002/oby.24213 (PMC11774011; doi:10.1002/oby.24213)
Supplement: Supplementary file 1 — Data S1. Supporting Information. [file OBY-33-385-s001.docx]

**Online supporting information for:**

**Interplay between genetic risk and built neighborhood conditions as predictor of BMI across the transition into adulthood**

Marthe de Roo^1^, MSc, Catharina Hartman^2^, PhD, Alfred Wagtendonk^3,4^, MSc, Hans W. Hoek^5,6,7^, PhD, Jeroen Lakerveld^3,4^, PhD, Tina Kretschmer^1^, PhD

**Affiliations:** ^1^Faculty of Behavioral and Social Sciences, Department of Pedagogy and Educational Sciences, University of Groningen, Groningen, the Netherlands; ^2^Interdisciplinary Center Psychopathology and Emotion Regulation (ICPE), University of Groningen, University Medical Center Groningen, Groningen, the Netherlands; ^3^Department of Epidemiology and Data Science, Amsterdam Public Health Research Institute, Amsterdam UMC, Vrije Universiteit Amsterdam, Amsterdam, the Netherlands; ^4^Upstream Team, www.upstreamteam.nl, Amsterdam UMC, Vrije Universiteit Amsterdam, Amsterdam, the Netherlands; ^5^Parnassia Psychiatric Institute, The Hague, the Netherlands; ^6^Department of Psychiatry, University of Groningen, University Medical Center Groningen, Groningen, the Netherlands; ^7^Department of Epidemiology, Columbia University, New York, USA

**Appendix A**

***Attrition Analyses***

We compared participants with complete data on all study variables (n=913, 33%) to those with at least one missing data point (n=1822, 67%) using Welch’s *t*-test for continuous variables and the chi-square test of independence for categorical variables. There were no significant differences between these groups in terms of the main predictor and outcome variables. With respect to the covariates, participants with complete data were younger at baseline (t_2008_=4.51, *P*<.001, Cohen’s *d*=0.19), more often female (*χ*^2^_1_=7.71, *P*=.005, Cramér’s *V*=0.05), and came from families with higher socioeconomic status (t_1920_=13.06, *P*<.001, Cohen’s *d*=0.52). They also had more mental health problems at baseline (t_1908_=-2.40, *P*=.02, Cohen’s *d*=0.11), were more likely to be from the high-risk cohort (*χ*^2^_1_=3.87, *P*=.05, Cramér’s *V*=0.04), lived in neighborhoods with higher socioeconomic status at ages 16 (t_2078_=3.77, *P*<.001, Cohen’s *d*=0.16), 19 (t_2106_=3.42, *P*<.001, Cohen’s *d*=0.14), and 25 (t_1873_=2.35, *P*=.02, Cohen’s *d*=0.11), and resided in more urban areas at age 25 (t_1817_=2.27, *P*=.02, Cohen’s *d*=0.11).

| **Variable** | **Mean (SD)**  **complete data (n=913)** | **Mean (SD)**  **incomplete data (n=1822)** |
| --- | --- | --- |
| Polygenic score for BMI | -0.02 (0.97) | 0.02 (1.03) |
| Age at baseline (2006) | 15.42 (1.49) | 15.71 (1.55) |
| Family socioeconomic status | 0.21 (0.74) | -0.19 (0.78) |
| Baseline mental health (2006) | 0.22 (0.21) | 0.24 (0.22) |
| BMI (2006, age 16) | 20.63 (3.12) | 20.95 (3.67) |
| BMI (2010, age 19) | 22.57 (3.76) | 22.67 (4.00) |
| BMI (2016, age 25) | 24.10 (4.33) | 24.24 (4.60) |
| Fast-food restaurant density (**Δ**2010-2006) | 0.77 (3.43) | 0.66 (3.51) |
| Fast-food restaurant density (**Δ**2016-2010) | 1.90 (6.23) | 1.46 (5.41) |
| Walkability (**Δ**2010-2006) | 3.80 (13.56) | 3.08 (14.30) |
| Walkability (**Δ**2015-2010) | 7.29 (21.84) | 6.20 (21.64) |
| Neighborhood SES 2006 (age 16) | -0.09 (1.00) | -0.26 (1.12) |
| Neighborhood SES 2010 (age 19) | -0.73 (1.32) | -0.93 (1.49) |
| Neighborhood SES 2016 (age 25) | -0.78 (1.41) | -0.93 (1.47) |
| Urban density 2006 (age 16) | 1304.85 (1115.22) | 1391.38 (1135.41) |
| Urban density 2011 (age 19) | 1760.46 (1602.56) | 1763.78 (1540.11) |
| Urban density 2016 (age 25) | 2686.50 (2252.72) | 2462.54 (2000.42) |
| Living situation 2006 (age 16) | 99.8% lives in family home | 99.3% lives in family home |
| Living situation 2010 (age 19) | 77.1% lives in family home | 76.9% lives in family home |
| Living situation 2016 (age 25) | 19.5% lives in family home | 22.7% lives in family home |
| Sex | 48.8% male | 54.6% male |
| Cohort | 21.7% high-risk cohort | 18.4% high-risk cohort |

**Appendix B**

***Genotyping and Imputation***

Blood samples were collected at T3 (T2 for the high-risk sample, combined n=1565). Participants who did not give blood were asked to provide buccal cells (Cytobrush®; n=360). DNA was extracted using a manual salting out procedure^1^ and stored at -80°C. Genotyping was performed using the Golden Gate Illumina BeadStation 500 and the Infinium™ HumanCytoSNP-12 v2.1 BeadChip platforms (Illumina Inc., San Diego, CA), according to the manufacturer’s protocols. DNA samples that were too heterogeneous, were duplicated or related, or were from non-European descent (as determined by principal components analysis of our samples combined with all 1000G samples) were excluded. In addition, genetic variants that had >5% missing data, minor allele frequency <1%, or deviated significantly from Hardy Weinberg equilibrium (p <10-6) were excluded. One SNP showed >5% mismatches and was excluded from the Golden Gate dataset after checking the minor allele frequency with HapMap. Genotypes were next imputed using the Haplotype Reference Consortium’s global reference panel on the Michigan Imputation server.^2,3^

We excluded one member of all sibling pairs (n=38), retaining the sibling for whom most data were available. If this was the same for both siblings, we retained the one with the highest subject ID number. This resulted in a total analytic sample of n=1676 for the regression analyses.

**References**

1. Miller SA, Dykes DD, Polesky HF. A simple salting out procedure for extracting DNA from human nucleated cells. *Nucleic Acids Res.* 1988;16(3):1215.

2. Das S, Forer L, Schönherr S, et al. Next-generation genotype imputation service and

methods. *Nat Genet.* 2016;48(10):1284-1287.

3. McCarthy S, Das S, Kretzschmar W, et al. A reference panel of 64,976 haplotypes for

genotype imputation. *Nat Genet.* 2016;48(10):1279-1283.

**Supplementary Figure S1.** Heatmap of correlations.

*Note.* PGS*_BMI_*=polygenic score for BMI, SES=socioeconomic status. n=2691. Note that missings may occur for specific comparisons. Correlations involving two binary variables are Phi coefficients and correlations involving a binary and a continuous variable are point-biserial correlations. All other correlations are Pearson’s *r* correlations.

^a^ 1=male, 2=female.

^b^ 1=population sample, 2=high-risk sample.

^c^ 1=living away from home, 2=living in family home.

**Supplementary Table S1.** Model fit indices for latent growth analyses of BMI. Growth is defined by intercept and linear slope.

|  |  | Chi-Square Test of Model Fit | |  |  | RMSEA | | |  |
| --- | --- | --- | --- | --- | --- | --- | --- | --- | --- |
| Model | AIC | Value (*df*) | *P* value | CFI | TLI | Estimate | 90% CI | *P* value | SRMR |
| Intercept-only | 43253.56 | 1216.57 (4) | <.001 | 0.34 | 0.51 | 0.33 | (0.32, 0.35) | <.001 | 0.49 |
| **Linear slope** | **40543.06** | **78.34 (1)** | **<.001** | **0.96** | **0.87** | **0.17** | **(0.14, 0.20)** | **0.00** | **0.05** |

*Note.* AIC=Akaike Information Criterion, *df*=degrees of freedom, CFI=Comparative Fit Index, TLI=Tucker-Lewis Index, RMSEA=Root Mean Square Error of Approximation, SRMR=Standardized Root Mean Square Residual. n=2735. Selected model is indicated in boldface.

**Supplementary Figure S2.** Estimated means of BMI across the study period based on the latent growth model.

*Note*. N=2735.

**Supplementary Table S2.** Sensitivity analysis: Standardized coefficients from models predicting BMI increase from the fast-food restaurant density difference scores, the polygenic score for BMI, and the fast-food restaurant density difference scores × polygenic score for BMI using 500m buffers.

|  |  | | **Intercept** | | **Slope** | |
| --- | --- | --- | --- | --- | --- | --- |
| **Model** | **Predictor** | | **β (SE)** | **95% CI** | **β (SE)** | **95% CI** |
| **1a. Main effect fast-food** | Fast-food restaurant density (**Δ** 2010-2006) | |  |  | -0.03 (0.03) | (-0.09, 0.03) |
| **restaurant density** | Fast-food restaurant density (**Δ** 2016-2010) | |  |  | 0.00 (0.03) | (-0.06, 0.05) |
| **difference score (n=2735)** | |  |  |  |  |  |
|  | |  |  |  |  |  |
| **1b. Fast-food restaurant** | Fast-food restaurant density (**Δ** 2010-2006) | |  |  | -0.03 (0.04) | (-0.10, 0.04) |
| **density difference score** | Fast-food restaurant density (**Δ** 2016-2010) | |  |  | 0.00 (0.03) | (-0.07, 0.07) |
| **× PGS*_BMI_* (n=1676)** | Polygenic score for BMI | | 0.32 (0.02)* | (0.28, 0.36) | 0.12 (0.03)* | (0.06, 0.17) |
|  | Fast-food restaurant density (**Δ** 2010-2006) × PGS*_BMI_* | |  |  | -0.02 (0.03) | (-0.08, 0.03) |
|  | Fast-food restaurant density (**Δ** 2016-2010) × PGS*_BMI_* | |  |  | -0.01 (0.03) | (-0.06, 0.04) |

*Note.* SE=standard error, CI=confidence interval, PGS*_BMI_*=polygenic score for BMI. Associations were adjusted for sex, age at baseline, cohort, family socioeconomic status, baseline mental health, and 20 principal components (model b) as time-invariant covariates, and living situation, neighborhood socioeconomic status, and urban density as time-varying covariates.

* indicates *p*<.05.

**Supplementary Table S3.** Sensitivity analysis: Standardized coefficients from models predicting BMI increase from the fast-food restaurant density difference scores, the polygenic score for BMI, and the fast-food restaurant density difference scores × polygenic score for BMI using 1500m buffers.

|  |  | | **Intercept** | | **Slope** | |
| --- | --- | --- | --- | --- | --- | --- |
| **Model** | **Predictor** | | **β (SE)** | **95% CI** | **β (SE)** | **95% CI** |
| **1a. Main effect fast-food** | Fast-food restaurant density (**Δ** 2010-2006) | |  |  | -0.01 (0.04) | (-0.09, 0.07) |
| **restaurant density** | Fast-food restaurant density (**Δ** 2016-2010) | |  |  | 0.01 (0.04) | (-0.07, 0.08) |
| **difference score (n=2735)** | |  |  |  |  |  |
|  | |  |  |  |  |  |
| **1b. Fast-food restaurant** | Fast-food restaurant density (**Δ** 2010-2006) | |  |  | 0.00 (0.05) | (-0.10, 0.10) |
| **density difference score** | Fast-food restaurant density (**Δ** 2016-2010) | |  |  | 0.01 (0.04) | (-0.07, 0.09) |
| **× PGS*_BMI_* (n=1676)** | Polygenic score for BMI | | 0.32 (0.02)* | (0.28, 0.36) | 0.12 (0.03)* | (0.06, 0.17) |
|  | Fast-food restaurant density (**Δ** 2010-2006) × PGS*_BMI_* | |  |  | -0.02 (0.03) | (-0.07, 0.04) |
|  | Fast-food restaurant density (**Δ** 2016-2010) × PGS*_BMI_* | |  |  | -0.01 (0.03) | (-0.07, 0.04) |

*Note.* SE=standard error, CI=confidence interval, PGS*_BMI_*=polygenic score for BMI. Associations were adjusted for sex, age at baseline, cohort, family socioeconomic status, baseline mental health, and 20 principal components (model b) as time-invariant covariates, and living situation, neighborhood socioeconomic status, and urban density as time-varying covariates.

* indicates *p*<.05.

**Supplementary Table S4.** Sensitivity analysis: Standardized coefficients from models predicting BMI increase from the walkability difference scores, the polygenic score for BMI, and the walkability difference scores × polygenic score for BMI using 500m buffers.

|  |  | | **Intercept** | | **Slope** | |
| --- | --- | --- | --- | --- | --- | --- |
| **Model** | **Predictor** | | **β (SE)** | **95% CI** | **β (SE)** | **95% CI** |
| **2a. Main effect** | Walkability (**Δ** 2010-2006) | |  |  | -0.04 (0.03) | (-0.10, 0.02) |
| **walkability difference** | Walkability (**Δ** 2015-2010) | |  |  | -0.03 (0.03) | (-0.09, 0.04) |
| **score (n=2735)** | |  |  |  |  |  |
|  | |  |  |  |  |  |
| **2b. Walkability** | Walkability (**Δ** 2010-2006) | |  |  | -0.06 (0.04) | (-0.13, 0.02) |
| **difference score** | Walkability (**Δ** 2015-2010) | |  |  | -0.03 (0.04) | (-0.10, 0.04) |
| **× PGS*_BMI_* (n=1676)** | Polygenic score for BMI | | 0.32 (0.02)* | (0.28, 0.36) | 0.11 (0.03)* | (0.06, 0.16) |
|  | Walkability (**Δ** 2010-2006) × PGS*_BMI_* | |  |  | -0.04 (0.03) | (-0.10, 0.02) |
|  | Walkability (**Δ** 2015-2010) × PGS*_BMI_* | |  |  | 0.01 (0.03) | (-0.04, 0.07) |

*Note.* SE=standard error, CI=confidence interval, PGS*_BMI_*=polygenic score for BMI. Associations were adjusted for sex, age at baseline, cohort, family socioeconomic status, baseline mental health, and 20 principal components (model b) as time-invariant covariates, and living situation, neighborhood socioeconomic status, and urban density as time-varying covariates.

* indicates *p*<.05.

**Supplementary Table S5.** Standardized coefficients from models predicting BMI increase from the walkability difference scores, the polygenic score for BMI, and the walkability difference scores × polygenic score for BMI using 1650m buffers.

|  |  | | **Intercept** | | **Slope** | |
| --- | --- | --- | --- | --- | --- | --- |
| **Model** | **Predictor** | | **β (SE)** | **95% CI** | **β (SE)** | **95% CI** |
| **2a. Main effect** | Walkability (**Δ** 2010-2006) | |  |  | -0.06 (0.05) | (-0.16, 0.03) |
| **walkability difference** | Walkability (**Δ** 2015-2010) | |  |  | -0.02 (0.05) | (-0.11, 0.07) |
| **score (n=2735)** | |  |  |  |  |  |
|  | |  |  |  |  |  |
| **2b. Walkability** | Walkability (**Δ** 2010-2006) | |  |  | -0.02 (0.06) | (-0.13, 0.09) |
| **difference score** | Walkability (**Δ** 2015-2010) | |  |  | -0.02 (0.05) | (-0.11, 0.08) |
| **× PGS*_BMI_* (n=1676)** | Polygenic score for BMI | | 0.31 (0.02)* | (0.27, 0.35) | 0.12 (0.03)* | (0.06, 0.18) |
|  | Walkability (**Δ** 2010-2006) × PGS*_BMI_* | |  |  | -0.02 (0.03) | (-0.08, 0.05) |
|  | Walkability (**Δ** 2015-2010) × PGS*_BMI_* | |  |  | 0.01 (0.03) | (-0.05, 0.07) |

*Note.* SE=standard error, CI=confidence interval, PGS*_BMI_*=polygenic score for BMI. Associations were adjusted for sex, age at baseline, cohort, family socioeconomic status, baseline mental health, and 20 principal components (model b) as time-invariant covariates, and living situation, neighborhood socioeconomic status, and urban density as time-varying covariates.

* indicates *p*<.05.

**Supplementary Table S6.** Complete case analysis: Standardized coefficients from models predicting BMI increase from the fast-food restaurant density difference scores, the polygenic score for BMI, and the fast-food restaurant density difference scores × polygenic score for BMI using 1000m buffers and only complete cases.

|  |  | **Intercept** | | **Slope** | |
| --- | --- | --- | --- | --- | --- |
| **Model** | **Predictor** | **β (SE)** | **95% CI** | **β (SE)** | **95% CI** |
| **1a. Main effect fast-food** | Fast-food restaurant density (**Δ** 2010-2006) |  |  | -0.02 (0.04) | (-0.10, 0.06) |
| **restaurant density** | Fast-food restaurant density (**Δ** 2016-2010) |  |  | 0.04 (0.04) | (-0.03, 0.11) |
| **difference score (n=1378)** |  |  |  |  |  |
|  |  |  |  |  |  |
| **1b. Fast-food restaurant** | Fast-food restaurant density (**Δ** 2010-2006) |  |  | -0.02 (0.04) | (-0.10, 0.07) |
| **density difference score** | Fast-food restaurant density (**Δ** 2016-2010) |  |  | 0.04 (0.04) | (-0.04, 0.11) |
| **× PGS*_BMI_* (n=1136)** | Polygenic score for BMI | 0.32 (0.03)* | (0.27, 0.37) | 0.15 (0.03)* | (0.09, 0.21) |
|  | Fast-food restaurant density (**Δ** 2010-2006) × PGS*_BMI_* |  |  | -0.03 (0.03) | (-0.09, 0.03) |
|  | Fast-food restaurant density (**Δ** 2016-2010) × PGS*_BMI_* |  |  | -0.01 (0.03) | (-0.07, 0.05) |

*Note.* SE=standard error, CI=confidence interval, PGS*_BMI_*=polygenic score for BMI. Associations were adjusted for sex, age at baseline, cohort, family socioeconomic status, baseline mental health, and 20 principal components (model b) as time-invariant covariates, and living situation, neighborhood socioeconomic status, and urban density as time-varying covariates.

* indicates *p*<.05.

**Supplementary Table S7.** Complete case analysis: Standardized coefficients from models predicting BMI increase from the walkability difference scores, the polygenic score for BMI, and the walkability difference scores × polygenic score for BMI using 1000m buffers and only complete cases.

|  |  | **Intercept** | | **Slope** | |
| --- | --- | --- | --- | --- | --- |
| **Model** | **Predictor** | **β (SE)** | **95% CI** | **β (SE)** | **95% CI** |
| **2a. Main effect** | Walkability (**Δ** 2010-2006) |  |  | -0.01 (0.05) | (-0.10, 0.09) |
| **walkability difference** | Walkability (**Δ** 2015-2010) |  |  | 0.04 (0.05) | (-0.06, 0.13) |
| **score (n=1378)** |  |  |  |  |  |
|  |  |  |  |  |  |
| **2b. Walkability** | Walkability (**Δ** 2010-2006) |  |  | -0.02 (0.05) | (-0.12, 0.09) |
| **difference score** | Walkability (**Δ** 2015-2010) |  |  | 0.02 (0.05) | (-0.08, 0.11) |
| **× PGS*_BMI_* (n=1136)** | Polygenic score for BMI | 0.32 (0.03)* | (0.27, 0.37) | 0.14 (0.03)* | (0.07, 0.20) |
|  | Walkability (**Δ** 2010-2006) × PGS*_BMI_* |  |  | 0.00 (0.03) | (-0.07, 0.06) |
|  | Walkability (**Δ** 2015-2010) × PGS*_BMI_* |  |  | 0.02 (0.03) | (-0.04, 0.08) |

*Note.* SE=standard error, CI=confidence interval, PGS*_BMI_*=polygenic score for BMI. Associations were adjusted for sex, age at baseline, cohort, family socioeconomic status, baseline mental health, and 20 principal components (model b) as time-invariant covariates, and living situation, neighborhood socioeconomic status, and urban density as time-varying covariates.

* indicates *p*<.05.
